# Enhanced photocatalytic degradation of toxic contaminants using Dy_2_O_3_-SiO_2_ ceramic nanostructured materials fabricated by a new, simple and rapid sonochemical approach

**DOI:** 10.1016/j.ultsonch.2021.105892

**Published:** 2021-12-24

**Authors:** Kamran Mahdavi, Sahar Zinatloo-Ajabshir, Qahtan A. Yousif, Masoud Salavati-Niasari

**Affiliations:** a Institute of Nano Science and Nano Technology, University of Kashan, Kashan P. O. Box. 87317-51167, Iran; b Department of Chemical Engineering, University of Bonab, P.O. Box. 5551761167 Bonab, Iran; c Department of Chemistry, College of Education, University of Al-Qadisiyah, Al Diwaniyah, Iraq

**Keywords:** Dysprosium oxide, SiO_2_, Nanostructure, Ultrasonic irradiation, Photocatalytic performance

## Abstract

The present study is on the fabrication of new photocatalytic nanocomposites (Dy_2_O_3_-SiO_2_) employing a basic agent, tetraethylenepentamine (Tetrene), through a simple, efficient and, quick sonochemical approach. The features of the fabricated photocatalytic nanocomposite were examined employing a variety of microscopic and spectroscopic methods such as XRD, EDS, TEM, FTIR, DRS, and FESEM. The outcomes of morphological studies demonstrated that by proper tuning of sonication time and ultrasonic power (10 min and 400 W), a porous nanocomposite composed of sphere-shaped nanoparticles with a particle size in the range of 20 to 60 nm could be fabricated. The energy gap for the binary Dy_2_O_3_-SiO_2_ nanophotocatalyst was determined to be 3.41 eV, making these nanocomposite favorable for removing contaminants. The photocatalytic performance of the optimal nanocomposite sample was tested for photodecomposition of several contaminants including erythrosine, thymol blue, eriochrome black T, Acid Red 14, methyl orange, malachite green, and Rhodamine B. The binary Dy_2_O_3_-SiO_2_ nanophotocatalyst exhibited superior efficiency toward the decomposition of the studied contaminants. It was able to degrade the erythrosine pollutant more effectively (92.9%). Optimization studies for the photocatalytic decomposition of each contaminant demonstrated that the best performance could be achieved at a specific amount of contaminant and nanocatalyst. Trapping experiments illustrated that hydroxyl radicals were more effectively involved in the decomposition of contaminant molecules by Dy_2_O_3_-SiO_2_ nanophotocatalyst.

## Highlights

• Introducing a facile sonochemical approach for the efficient preparation of new photocatalytic nanocomposites (Dy_2_O_3_-SiO_2_).
• Remarkable effect of SiO_2_ on improving photocatalytic performance of Dy_2_O_3_.
• Excellent photocatalytic performance of sonochemically fabricated Dy_2_O_3_-SiO_2_ nanocomposite in removal of organic pollutants under sunlight, for the first time.
• Enhanced optical absorption and having a high specific surface area were responsible for enhanced photocatalytic performance.
• Significant effect of sonication on the synthesis of high-performance binary Dy_2_O_3_-SiO_2_ nanophotocatalys.

## Graphical abstract

**Figure:**
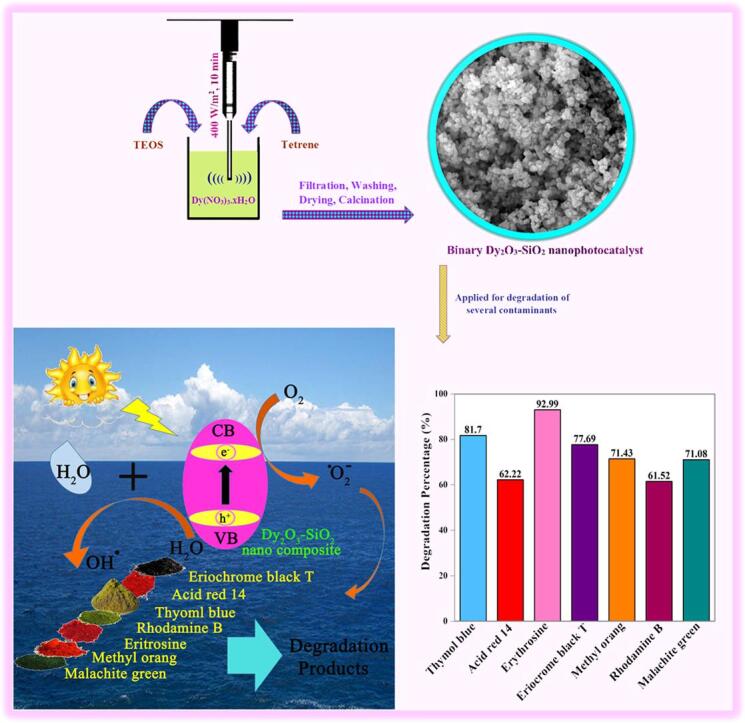

## 1 Introduction

Rapid population growth, along with the emergence of various industries, has led to the discharge of effluents containing toxic organic contaminants such as various dyes into the environment [1], [2]. Release of effluents containing toxic organic compounds such as erythrosine [3], thymol blue [4], eriochrome black T [5], Acid Red 14 [6], methyl orang [7], malachite green [8], and Rhodamine B [9] can have adverse impacts on the environment and human life, as these organic compounds have poor biodegradability [10], [11], [12]. Among the various solutions reported so far, photocatalysis can be beneficial through the direct usage of solar energy to decompose and remove toxic contaminants, including dyes [13], [14], [15]. The solar photocatalytic process does not require any additional chemical agents or the usage of any other energy to treat the contaminated water [16], [17], [18]. Thus, with the increasing demand for the decomposition and elimination of toxic and unsafe contaminants, the design and development of high-performance nanostructured photocatalysts have become one of the most popular research topics [19], [20], [21]. So far, a wide range of nanostructures and nanocomposites have been explored to treat water contaminated with organic compounds [22], [23], [24]. Nevertheless, there is still a strong need to design and produce new high-efficiency photocatalytic compounds through reproducible and large-scale approaches.

Dysprosium oxide with the formula Dy_2_O_3_ is a compound based on rare earth elements that has special features that are derived from their 4f electrons [25]. It is a basic substance, with exceptional thermal stability, and high insolubility properties [26], [27]. This oxide compound crystallizes in three different phases: cubic, hexagonal, and monoclinic [28]. What is more, dysprosium oxide and compounds based on this oxide have unique features, making their wide use in luminescent compounds [29], [30], catalysis [31], MRI contrast agents [32], fuel cells [33], and ceramics [34] possible. Since Dy_2_O_3_ has a wide energy gap (4.8 eV) [35], efforts have been made to improve its photocatalytic ability by doping it with other compounds, such as zinc oxide, which tunes its energy gap [36]. Up to present, a variety of methods have been reported for the fabrication of dysprosium oxide structures, including precipitation [25], thermal decomposition [37], [38], combustion [30], sol–gel process [26], and hydrothermal [39]. The methods employed so far to fabricate nanostructured Dy_2_O_3_ have drawbacks such as high time and energy consumption, the need for costly precursors, and multiple and sometimes complex steps [26], [37], [38]. The use of ultrasound waves to fabricate a variety of nanostructures is an area of interest to many scientists because it has significant benefits [40]. Ultrasonic radiation accelerates the reactions because high temperatures as well as high pressures are fabricated in the reaction medium, which causes the reactions to take place in a short time [41]. In addition, low energy consumption, good ability to tune the dimensions, architecture, and morphology of different compounds, and simplicity are other unique features of the use of sonochemistry, which has made it very popular for the fabrication of nanostructured compounds [42].

In order to eliminate the shortcomings including secondary contamination owing to high dispersion of nanostructured catalysts in the aqueous medium or the impossibility of effective binding of contaminant molecules to larger photocatalyst clusters, the combination of photocatalysts with adsorbents has been considered as a desirable approach [43]. In the nanocomposite formed, the adsorbent component will efficiently adsorb the contaminant molecules and the nanophotocatalyst component with active sites will cause the decomposition of the contaminant molecules. So far, silicon dioxide has been employed as a support to improve the performance of titanium dioxide catalyst [44].

Very few reports are available for the fabrication of Dy_2_O_3_-SiO_2_ nanocomposites, which have drawbacks such as high energy consumption as well as a long process [45]. The present study on the fabrication of new photocatalytic nanocomposites (Dy_2_O_3_-SiO_2_) employing a basic agent, tetraethylenepentamine (Tetrene), through a simple, efficient, and quick sonochemical approach. The combination of silicon dioxide with a porous structure with Dy_2_O_3_ can both form a nanocomposite photocatalyst with a narrower energy gap and enhance the rate of adsorption of pollutant molecules. To the best of the author's knowledge, no previous experimental research has focused on the efficient production of Dy_2_O_3_-SiO_2_ nanocomposites using ultrasonic waves and Tetrene and the study of its photocatalytic efficiency in the decomposition of several toxic contaminants. The effect of altering the sonication time and ultrasonic power on the architecture, dimensions, and morphology of photocatalytic nanocomposite was explored to select the optimal nanostructure. Furthermore, in order to achieve the best photocatalytic efficiency in the decomposition of each contaminant, the effects of the amount of contaminant as well as the dose of the composite nanostructure were investigated.

## 2 Experimental

### 2.1 Materials and characterization

All reagents employed in this experimental study, including dysprosium (III) nitrate, tetraethylenepentamine (Tetrene), and Si(OC₂H₅)₄ (TEOS), were of analytical purity and were all purchased from Merck. A diffractometer of Philips Company was employed to record the X-ray diffraction (XRD) outcome of the prepared nanocomposite photocatalyst. Morphological studies and chemical composition analysis of the produced nanocomposite samples were performed by a field emission scanning electron microscopy (MIRA3 FEG-SEM) coupled with energy-dispersive X-ray spectroscopy (EDS). A UV–visible spectrophotometer (Shimadzu, UV-2550, Japan) was employed to examine the optical absorption feature of the fabricated Dy_2_O_3_-SiO_2_ nanocomposite. The morphology and structure of the fabricated nanocatalyst were examined in more detail with a transmission electron microscopy (TEM, FEI F20). Fourier Transform Infrared Spectroscopy (FTIR) investigation was performed with a Magna-IR, spectrometer 550 Nicolet.

### 2.2 Preparation of binary Dy_2_O_3_-SiO_2_ nanophotocatalyst

**Scheme 1:**
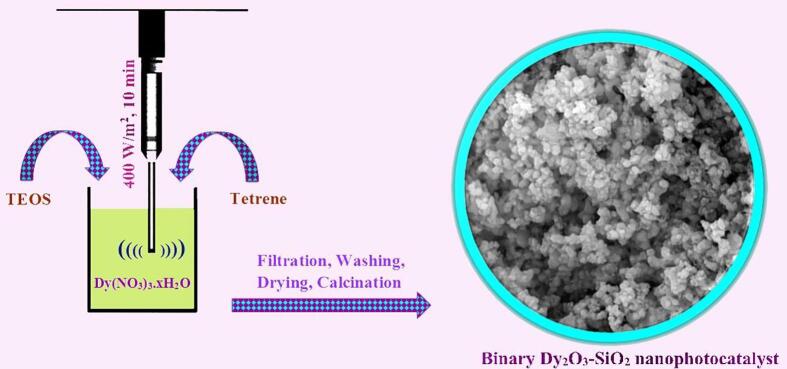
Schematic diagram illustrating the production route of binary Dy_2_O_3_-SiO_2_ nanocomposite (sample 5).

A simple, efficient, and quick sonochemical approach was employed to produce binary Dy_2_O_3_-SiO_2_ nanophotocatalyst. First, 1 mmol of TEOS was dissolved in ethanol, and then it was added dropwise to a solution containing 1 mmol of dysprosium nitrate dissolved in ethanol. Then the pH of the resulting mixture was tuned to about 10 with the help of a new alkaline agent (Tetrene), and it was irradiated with the ultrasonic probe at 400 W for 10 min. The resulting precipitate was then dried after washing several times with ethanol. Binary Dy_2_O_3_-SiO_2_ nanophotocatalyst (sample 5) was fabricated by calcination of the residual mass at 1000 °C within 8 h (see Scheme 1). The effect of change in the sonication time and ultrasonic power on the architecture, dimensions, and morphology of binary Dy_2_O_3_-SiO_2_ nanophotocatalyst was explored (see Table 1).

**Table 1:** Reaction conditions for sonochemical synthesis of all samples.

**Sample no**	**Dy:Si ratio**	**Basic agent**	**Sonication time (min)**	**Sonication power (W)**	**Figure of FESEM images**
1	1:1	Tetrene	10	160	1a and b
2	1:1	Tetrene	15	160	1c and d
3	1:1	Tetrene	20	160	1e and f
4	1:1	Tetrene	10	280	2a and b
5	1:1	Tetrene	10	400	2c and d

### 2.3 Photocatalytic performance of binary Dy_2_O_3_-SiO_2_ nanocatalyst

The photocatalytic performance of the binary Dy_2_O_3_-SiO_2_ nanophotocatalyst was tested for photodecomposition of several contaminants, including erythrosine, thymol blue, eriochrome black T, Acid Red 14, methyl orange, malachite green, and Rhodamine B. In each test, a certain amount of the binary Dy_2_O_3_-SiO_2_ nanophotocatalyst was dispersed in 50 ml of a solution comprising a specified quantity of the target contaminant. Then the resulting suspension was stirred in the darkness for half an hour to establish adsorption–desorption equilibrium [18]. The light source (400 W mercury lamp) was then switched on, and the suspension irradiated [46]. The efficiency of the sonochemically prepared Dy_2_O_3_-SiO_2_ nanophotocatalyst was evaluated and reported with ٪efficiency = (A_t_ /A_0_) × 100 [18]. A_0_ and A_t_ signify the absorbance of each contaminant before and after light irradiation [18].

## 3 Results and discussion

### 3.1 FESEM studies

**Fig. 1:**
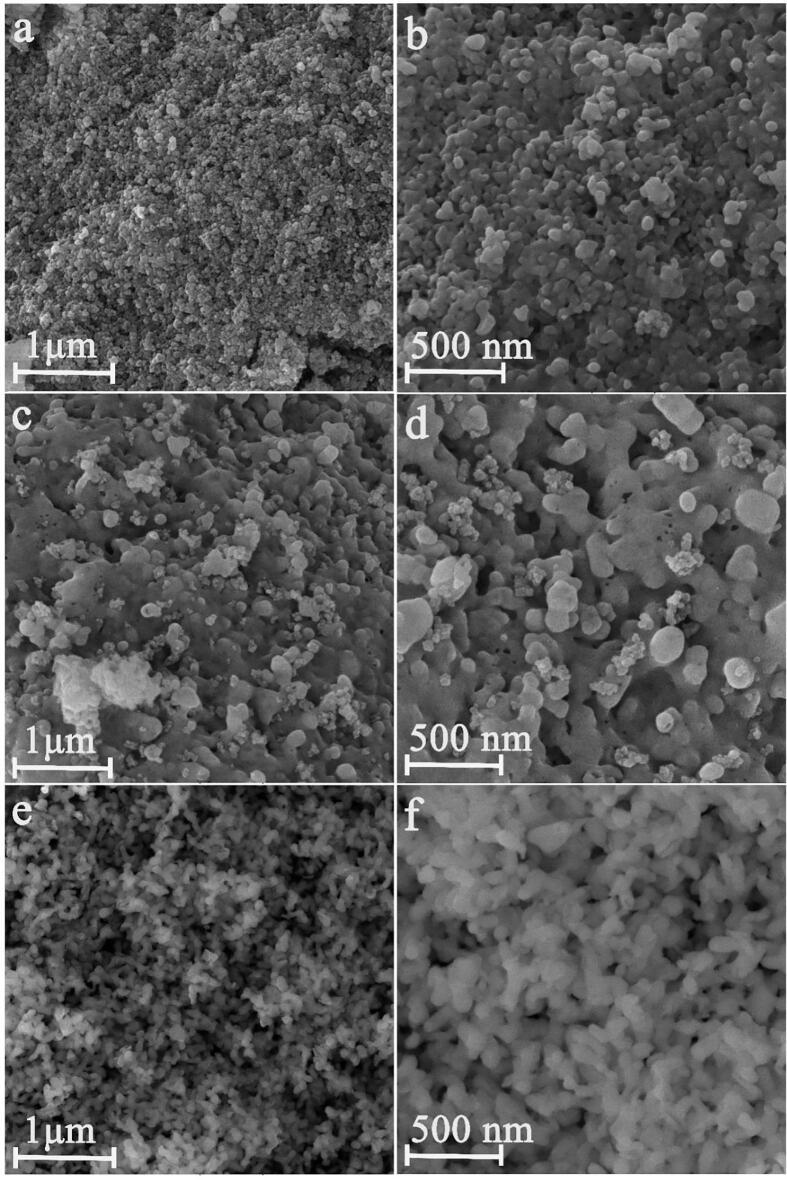
FESEM images of composite samples 1, 2, and 3 fabricated by sonication for 10 (a and b), 15 (c and d), and 20 (e and f) minutes.

**Fig. 2:**
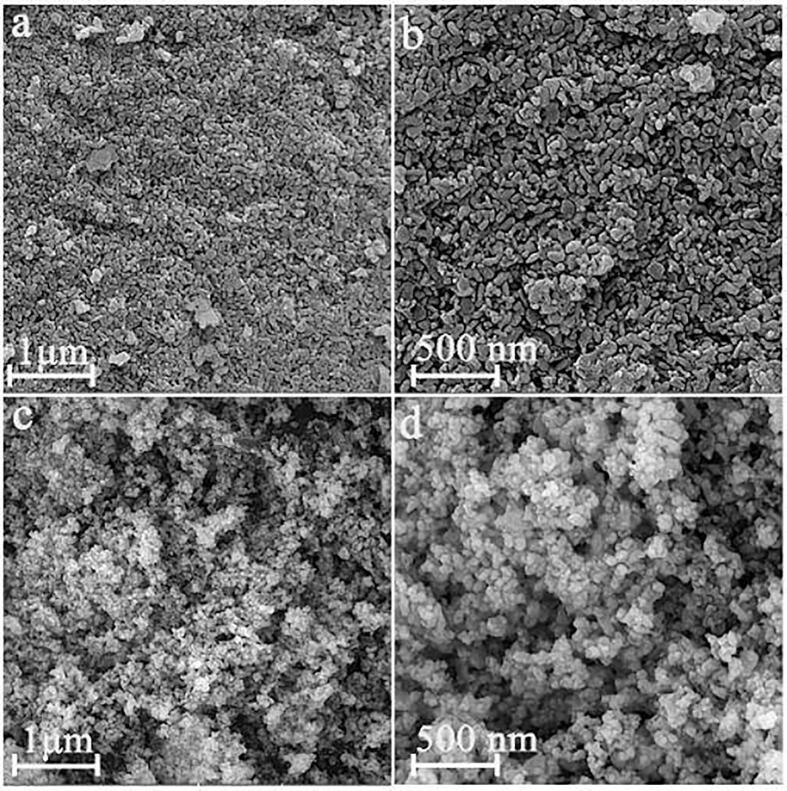
FESEM images of composite samples 4 and 5 made with 280 (a and b) and 400 (c and d) W ultrasonic power.

In this experimental work, a new alkaline agent, tetraethylenepentamine (Tetrene) was utilized to prepare the binary Dy_2_O_3_-SiO_2_ nanocomposite via a quick sonochemical approach. First, instrumental variables, including tuning sonication time as well as ultrasound power, were tuned to determine the proper conditions for creating a nanostructured product with favorable features in terms of uniformity and particle size (see Fig. 1, Fig. 2). FESEM outcomes for composite samples 1, 2, and 3 fabricated by sonication for 10, 15, and 20 min are displayed in Fig. 1. By sonication for 10 min, sphere-shaped nanoparticles were fabricated that are relatively uniform (Fig. 1a and b). With sonication longer than 10 min, the tendency of the prepared nanoparticles to aggregate enhanced, and irregular micro/nanostructures were formed (Fig. 1c–f). It seems that nanoparticles that have been fabricated in a shorter time owing to their high surface energy can act as initial nuclei and have grown through the Ostwald process, resulting in irregular micro/nanostructures [47], [48]. Enhancing the sonication time to 20 min also did not have a positive effect on improving the uniformity of the nanostructures and diminishing the particle size. It led to the creation of irregularly shaped nanostructures. It seems that with the prolongation of sonication, the effect of the Ostwald process has been greater than the mechanical effects of ultrasound waves. As a result, it has enhanced the particle size [48], [49]. Therefore, it can be concluded that sonication for 10 min can be proper for the preparation of sphere-shaped Dy_2_O_3_-SiO_2_ nanoparticles.

FESEM outcomes for composite samples 4 and 5 made with 280 and 400 W ultrasonic power are exhibited in Fig. 2. By enhancing the ultrasonic power from 160 W (Fig. 1a and b) to 280 (Fig. 2a and b) and then 400 W (Fig. 2c and d), the accumulation and aggregation of nanoparticles diminishes, the uniformity improves, and the particle size reduces. It can be seen that sphere-shaped composite nanoparticles with excellent uniformity and also tinier particle size are fabricated by tuning the ultrasound power to 400 W. Hence, enhancing the ultrasonic power remarkably affects the uniformity of the composite nanostructure as well as its architecture and particle size. Applying ultrasonic power above 160 W seems to accelerate the collapse of the cavitation bubbles, bringing in a stronger shock wave that hinders nanoparticles from accumulating more effectively [50]. So, the outcomes of morphological studies demonstrate that by proper tuning of sonication time and ultrasonic power (10 min and 400 W), a porous nanocomposite composed of sphere-shaped nanoparticles with a narrow size distribution can be fabricated. This composite nanostructure was selected for further investigation as well as photocatalytic decomposition of various contaminants.

### 3.2 Formation mechanism of sphere-shaped Dy_2_O_3_-SiO_2_ nanoparticles

Effective help to control the architecture of various nanostructures has been reported to be one of the beneficial applications of ultrasound waves. Desirable and distinctive nanoscale structures with great uniformity can be fabricated by the cavitation fabricated by the application of sonication [50]. According to the hot-spot theory, the conversion of huge structures into tiny particles can be easily facilitated and accelerated by creating very high temperatures as well as releasing enormous amounts of energy that occur during the collapse of bubbles [50]. So, as the above outcomes showed, tuning the ultrasonic radiation conditions can be advantageous in controlling the composite nanostructure architecture. Due to the adsorption of ultrasonic waves by water molecules, hydroxyl radical species are fabricated that can play an efficient role in the hydrolysis of dysprosium and silicon precursors and thus have a substantial contribution to the sonochemical fabrication of composite nanophotocatalyst (Dy_2_O_3_-SiO_2_) [51]. The possible mechanism for sonochemical creation of sphere-shaped Dy_2_O_3_-SiO_2_ nanoparticles can be summarized as follows [42], [52]:

H_2_O + ultrasound waves → H^.^ + OH^.^

OH^.^ + H_2_N(CH_2_CH_2_NH)_3_CH_2_CH_2_NH_2_ + 2H_2_O → H_3_N^+^(CH_2_CH_2_NH)_3_CH_2_CH_2_N^+^H_3_ + 2OH^–^ + by products

3OH^–^ + Dy(NO_3_)_3_ → 3NO_3_^–^+ Dy(OH)_3_

Dy(OH)_3_ Dy_2_O_3_

TEOS + H_2_O + OH^–^ → Si-(OH)_4_ + by-product

2Si-(OH)_4_ → SiO_2_ + by-product

Dy_2_O_3_ + SiO_2_ → Dy_2_O_3_/SiO_2_[42], [52]

### 3.3 TEM, EDS, XRD, and FTIR studies

**Fig. 3:**
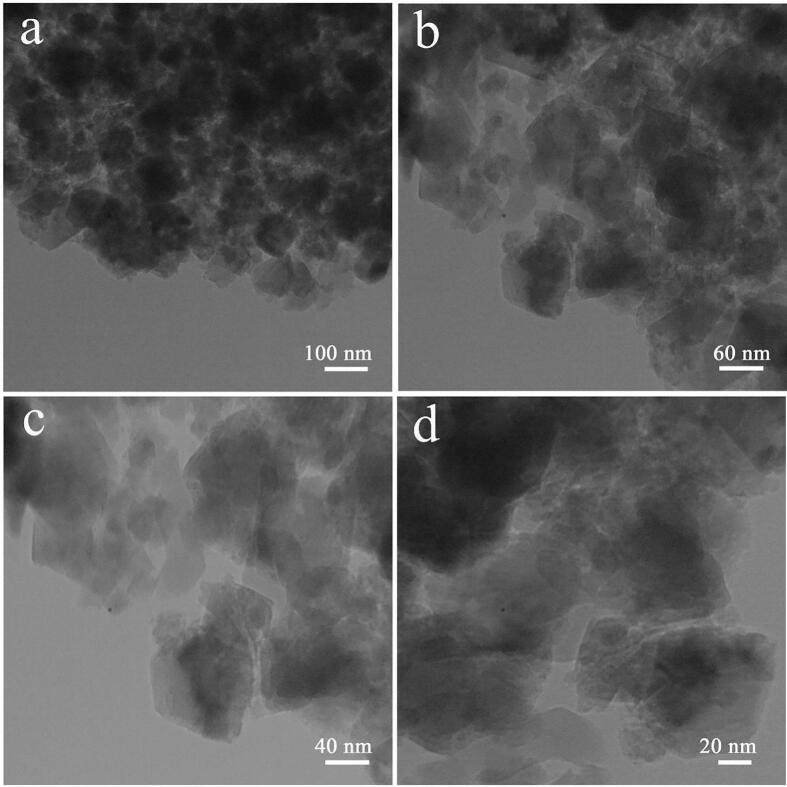
TEM images of binary Dy_2_O_3_-SiO_2_ nanocomposite (sample 5).

**Fig. 4:**
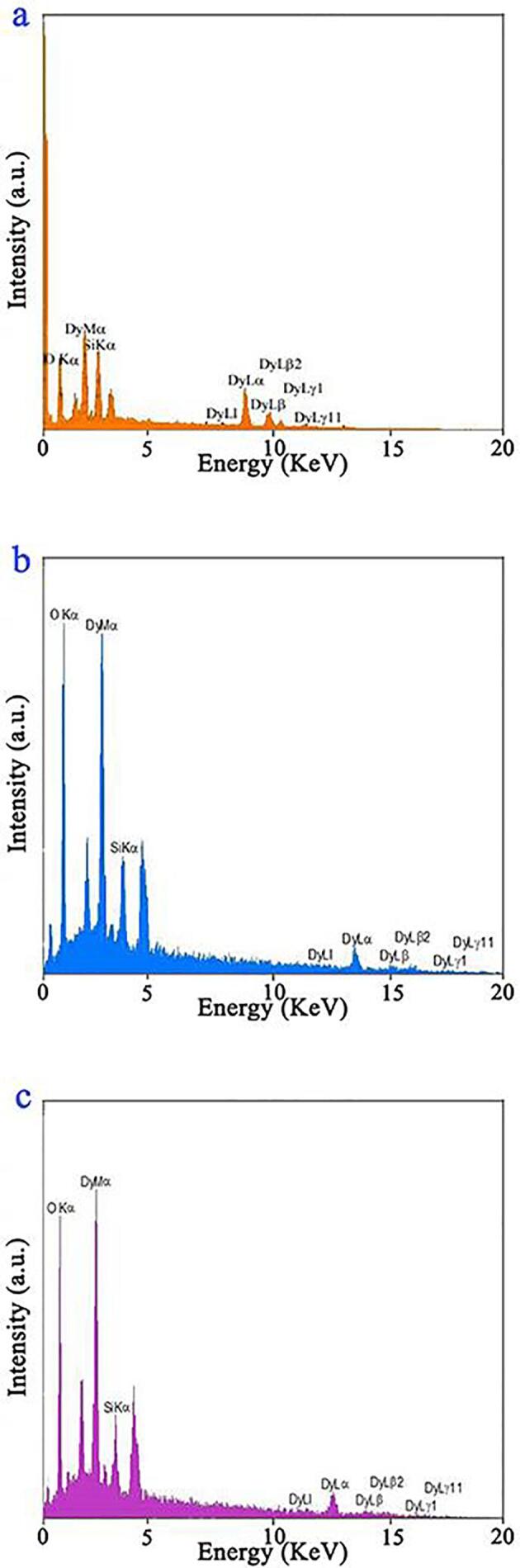
EDS patterns of samples 1 (a), 4 (b), and 5 (c).

TEM was utilized for the sake of minutely probing the architecture and microstructure of the selected composite nanostructure (Fig. 3). The sonochemically fabricated oxide sample consists of approximately sphere-shaped particles with a particle size in the range of 20 to 60 nm. The elemental composition of the samples fabricated via sonochemistry in three different ultrasonic powers, samples 1, 4, and 5 was determined by EDS. In Fig. 4, only the signals of the elements oxygen, silicon, and dysprosium can be clearly seen, corroborating their presence and the purity of the nanostructures.

**Fig. 5:**
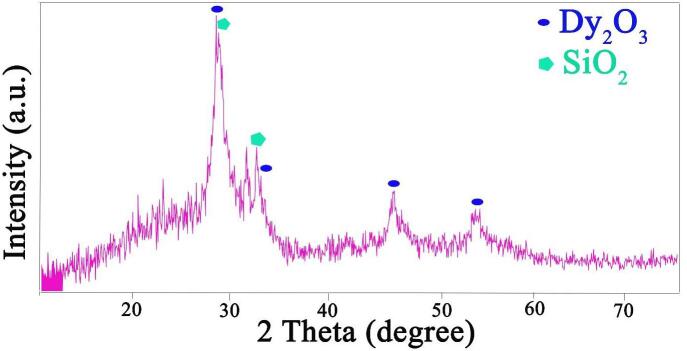
XRD pattern of binary Dy_2_O_3_-SiO_2_ nanocomposite (sample 5).

As an efficient and exact tool, XRD was employed to evaluate the crystal phase of the selected sample (Fig. 5). The characteristic diffraction lines of cubic Dy_2_O_3_ (JCPDS no. 01-074-1985) and tetragonal SiO_2_ (JCPDS no. 00-047-1300) can be found in Fig. 5, which manifests the fabrication of the binary Dy_2_O_3_-SiO_2_ nanocomposite with poor crystallization. The crystal size of the selected Dy_2_O_3_-SiO_2_ nanostructure was estimated to be about 17.4 nm employing the Scherrer formula [50]. No signal indicative of the impurity was observed in XRD profile, signifying the binary nanocomposite is pure.

**Fig. 6:**
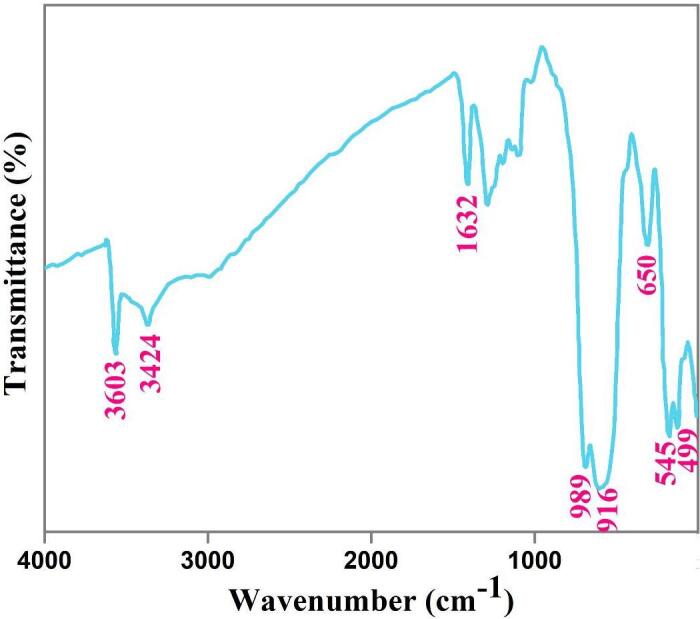
FT-IR spectrm of binary Dy_2_O_3_-SiO_2_ nanocomposite (sample 5).

The selected sample was also examined by FTIR to further corroborate the fabrication of composite nanophotocatalyst (Dy_2_O_3_-SiO_2_). Signals around 990, 916, and 650 cm^−1^ correspond to Si-O-Si bonds that manifest the creation of the SiO_2_ phase (see Fig. 6) [53], [54]. Characteristic signals of Dy_2_O_3_ are observed near 545 and 499 cm^−1^ [31]. The absorption signals at 3603, 3424, and 1632 cm^−1^ demonstrate the physisorbed water molecules [41]. Thus, FTIR findings are in line with XRD and EDS outcomes.

### 3.4 Optical and textural properties of sphere-shaped Dy_2_O_3_-SiO_2_ nanoparticles

**Fig. 7:**
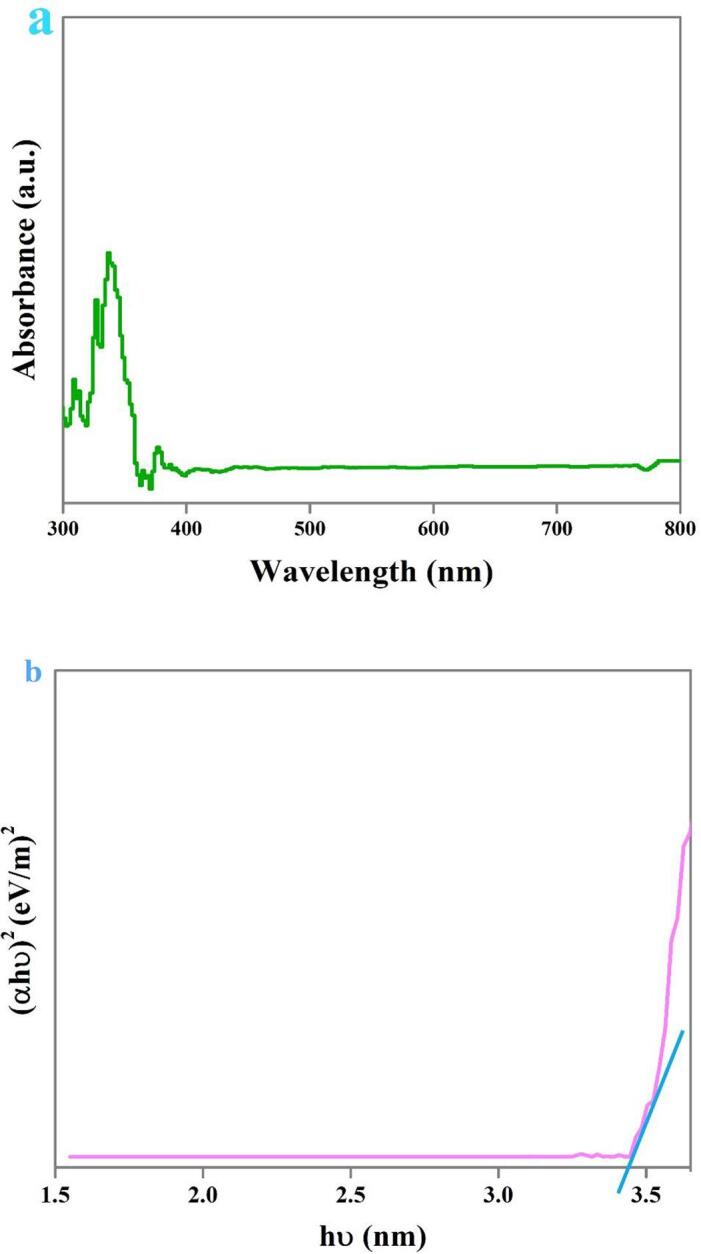
DRS spectrm (a), plot to determine the band gap (b) of binary Dy_2_O_3_-SiO_2_ nanocomposite (sample 5).

The optical absorption features of the selected composite nanostructure were explored by DRS (see Fig. 7). The binary Dy_2_O_3_-SiO_2_ nanocomposite exhibits an absorption edge at 367 nm. The energy gap of about 3.41 eV for the selected binary nanocomposite was determined employing DRS outcomes and Tauc’s formula [18]. The sonochemically fabricated binary Dy_2_O_3_-SiO_2_ nanocomposite has a narrower energy gap than the pure dysprosium oxide previously reported (4.8 eV) [35]. It can be concluded that the addition of silicon dioxide into Dy_2_O_3_ effectively tuned its energy gap, and the prepared binary Dy_2_O_3_-SiO_2_ nanocomposite could possibly manifest improved photocatalytic performance in removing contaminants.

**Fig. 8:**
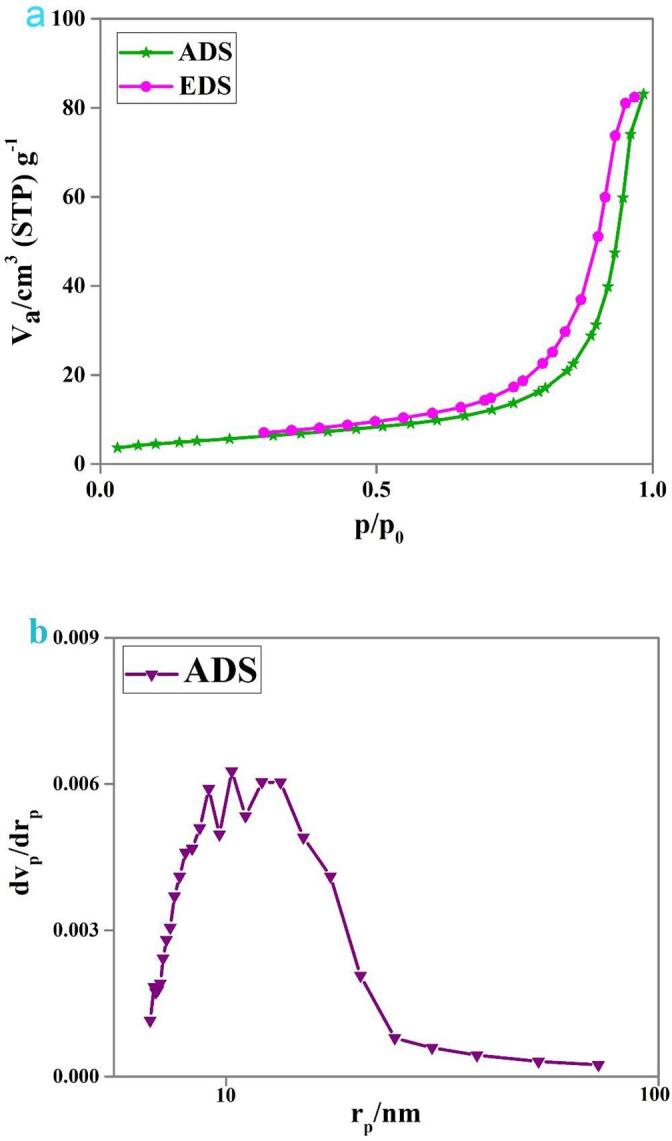
N_2_ adsorption/desorption isotherm (a) and pore size distribution curve (b) of binary Dy_2_O_3_-SiO_2_ nanocomposite (sample 5).

The textural features of the sonochemically fabricated binary Dy_2_O_3_-SiO_2_ nanocomposite were also examined, and the outcomes are presented in Fig. 8 and Table 2. It can be seen that the prepared binary nanocomposite sample is mesoporous in nature. The compounds with the great specific surface area can more efficiently adsorb contaminant molecules and also provide a large number of active sites for the decomposition of contaminant, resulting in excellent photocatalytic efficiency [55]. With a good specific surface area, it can be expected that binary nanocomposites can denote outstanding photocatalytic performance.

**Table 2:** The textural properties of the binary Dy_2_O_3_-SiO_2_ nanocomposite (sample 5).

Sample no	BET area (m^2^g^−1^)	Pore volume (cm^3^ g^−1^)	Pore diameter (nm)
5	19.847	0.1285	25.904

### 3.5 Evaluation of the photocatalytic activity of binary Dy_2_O_3_-SiO_2_ nanocomposite

**Fig. 9:**
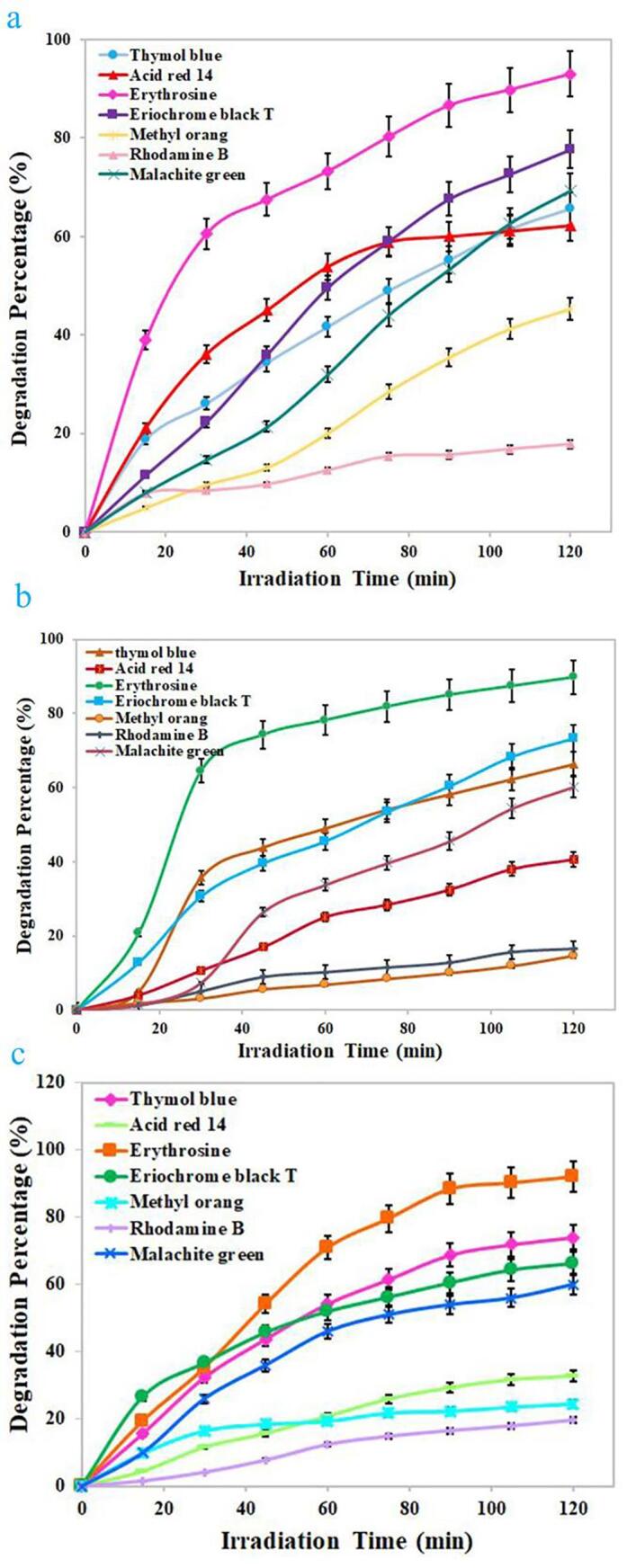
Photocatalytic degradation of various pollutants with different concentrations of 5 (a), 10 (b), and 15 (c) ppm in the presence of 0.015 g binary Dy_2_O_3_-SiO_2_ nanocomposite (sample 5), under UV light irradiation.

**Fig. 10:**
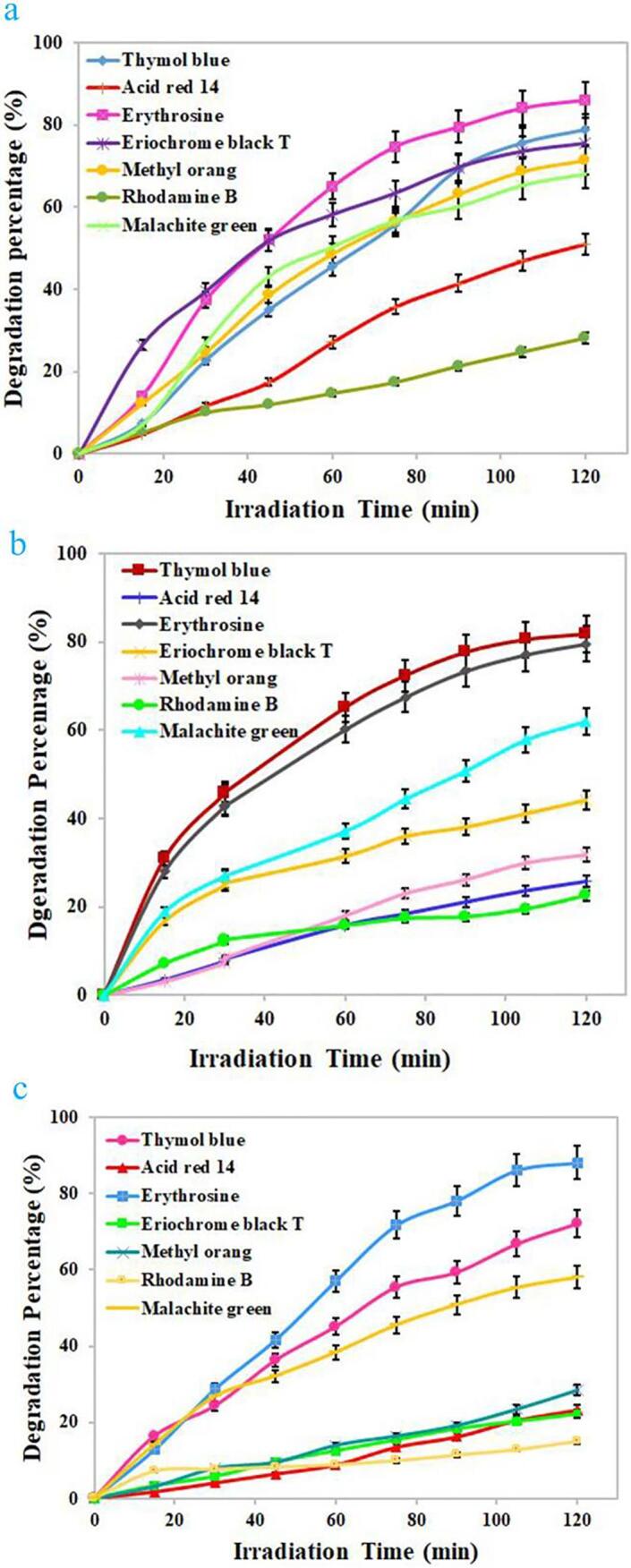
Photocatalytic degradation of various pollutants with different concentrations of 5 (a), 10 (b), and 15 (c) ppm in the presence of 0.03 g of binary Dy_2_O_3_-SiO_2_ nanocomposite (sample 5), under UV light irradiation.

**Fig. 11:**
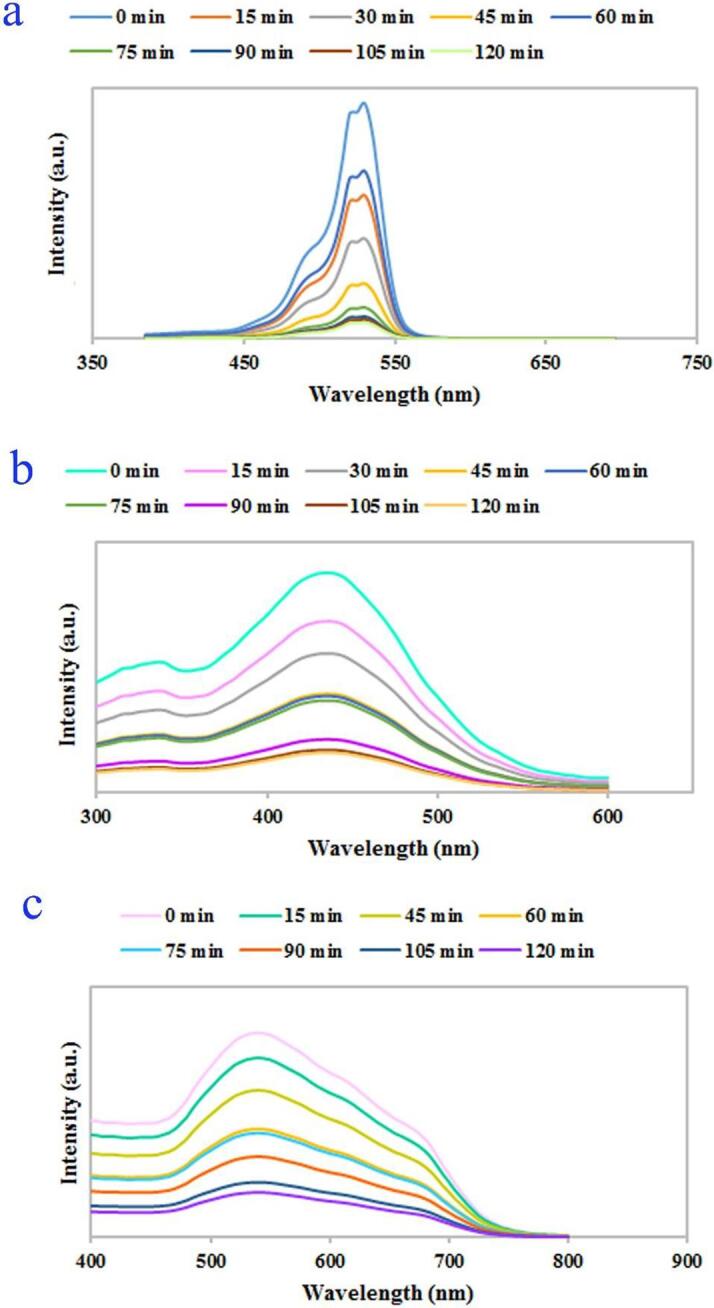
UV–vis absorption spectra depicting decomposition of (a) erythrosine (0.015 g of catalyst, concentration of 5 ppm of dye), (b) thymol blue (0.03 g of catalyst, concentration of 10 ppm of dye), and (c) eriochrome black T (0.015 g of catalyst, concentration of 5 ppm of dye) under UV light irradiation.

Due to the proper energy gap of the selected composite nanostructure, its photocatalytic performance was tested under ultraviolet light for photodecomposition of water contaminant. Several dyes including erythrosine, thymol blue, eriochrome black T, Acid Red 14, methyl orange, malachite green, and Rhodamine B were selected as target contaminants to test the performance of the binary Dy_2_O_3_-SiO_2_ nanocatalyst. In order to achieve the greatest efficiency in photocatalytic decomposition of each contaminant, the impact of variables including contaminant concentration and quantity of Dy_2_O_3_-SiO_2_ nanophotocatalyst was explored (see Fig. 9, Fig. 10). Solutions with different concentrations of 5, 10, and 15 ppm were employed as target contaminants. Various quantities of binary Dy_2_O_3_-SiO_2_ nanophotocatalyst, 0.015 and 0.03 g, were applied for photocatalytic testing. Decomposition of any contaminants was negligible without UV light or without the usage of binary Dy_2_O_3_-SiO_2_ nanocatalyst. Thus, the presence of both factors was necessary to decompose each of the pollutants. It can be seen that the studied variables, the concentration of the contaminant solution and the quantity of Dy_2_O_3_-SiO_2_ nanophotocatalyst, both significantly affect the percentage of decomposition. The effect of these variables for the percentage of decomposition of each contaminant is different from other contaminants. By increasing the quantity of binary Dy_2_O_3_-SiO_2_ nanocomposite from 0.015 g to 0.03, in the case of contaminants with a concentration of 5 ppm, the percentage of decomposition of all 4 contaminants was enhanced (Fig. 9, Fig. 10a). It can be observed in the case of contaminant with a concentration of 5 ppm, employing 0.03 g of binary Dy_2_O_3_-SiO_2_ nanocatalyst, 78.75, 71.43, 28.16, and 68.08% of thymol blue, methyl orange, Rhodamine B, and malachite green were decomposed after 120 min of UV exposure (Fig. 10a). In contrast, the percentage of decomposition of Acid Red 14, erythrosine, and eriochrome black T contaminant diminished to 51.04, 86.11, and 75.45 %. For contaminants with a concentration of 10 ppm, by altering the amount of binary Dy_2_O_3_-SiO_2_ nanocatalyst from 0.015 g to 0.03 g, the decomposition percentage of thymol blue, methyl orange, Rhodamine B, and malachite green enhanced to 81.70, 31.81, 20.48, and 61.98 (Fig. 9, Fig. 10b). In comparison, the percentage of decomposition of Acid Red 14, erythrosine, and eriochrome black T contaminant diminished to 25.64, 78.59, and 44.16%. In the case of contaminants with a concentration of 15 ppm, utilizing 0.015 g of binary Dy_2_O_3_-SiO_2_ nanocomposite, 73.97, 32.65, 92.05, 66.19, 29.42, 19.05, and 60% of thymol blue, Acid Red 14, erythrosine, eriochrome black T, methyl orange, Rhodamine B, and malachite green were removed after 120 min of UV exposure. Applying 0.03 g of the composite nanostructure, a lower percentage of decomposition was observed for all contaminants (Fig. 9, Fig. 10c). Based on the above outcomes, it can be concluded that the concentration of 5 ppm of contaminant, as well as the quantity of 0.015 g of Dy_2_O_3_-SiO_2_ nanocatalyst, is the most appropriate conditions to have the highest percentage of degradation of Acid Red 14, erythrosine, and eriochrome black T contaminants (Fig. 9a). In contrast, the highest percentage of decomposition of methyl orange, Rhodamine B, and malachite green occurred under optimal conditions, including a concentration of 5 ppm of contaminant and 0.03 g of Dy_2_O_3_-SiO_2_ nanocatalyst (Fig. 10a). In the case of thymol blue, conditions including 10 ppm of contaminant and 0.03 g of Dy_2_O_3_-SiO_2_ nanocomposite were more proper to achieve the highest percentage of decomposition (Fig. 10b). Under optimized conditions, the binary Dy_2_O_3_-SiO_2_ nanophotocatalyst exhibited superior efficiency toward the decomposition of the studied contaminants, and the highest percentage of erythrosine contaminant (about 92.99) was decomposed. The addition of silicon dioxide into Dy_2_O_3_ results in the formation of a binary Dy_2_O_3_-SiO_2_ nanocomposite with a good specific surface area that can be very beneficial for efficient absorption of light as well as enhancing the adsorption of target contaminant molecules. It also tunes its energy gap, which results in more separation of the charge carrier [56], [57]. For the above reasons, it seems that sonochemically prepared Dy_2_O_3_-SiO_2_ nanocatalyst manifest superior efficiency in the decomposition of pollutants. Fig. 11a–c exhibit UV–vis absorption spectra of erythrosine, eriochrome black T, and thymol blue with respect to time over the binary Dy_2_O_3_-SiO_2_ nanocomposite, which illustrates that the absorption intensity diminishes as illumination time enhances and; the decomposition of all three contaminant occurs continuously.

**Fig. 12:**
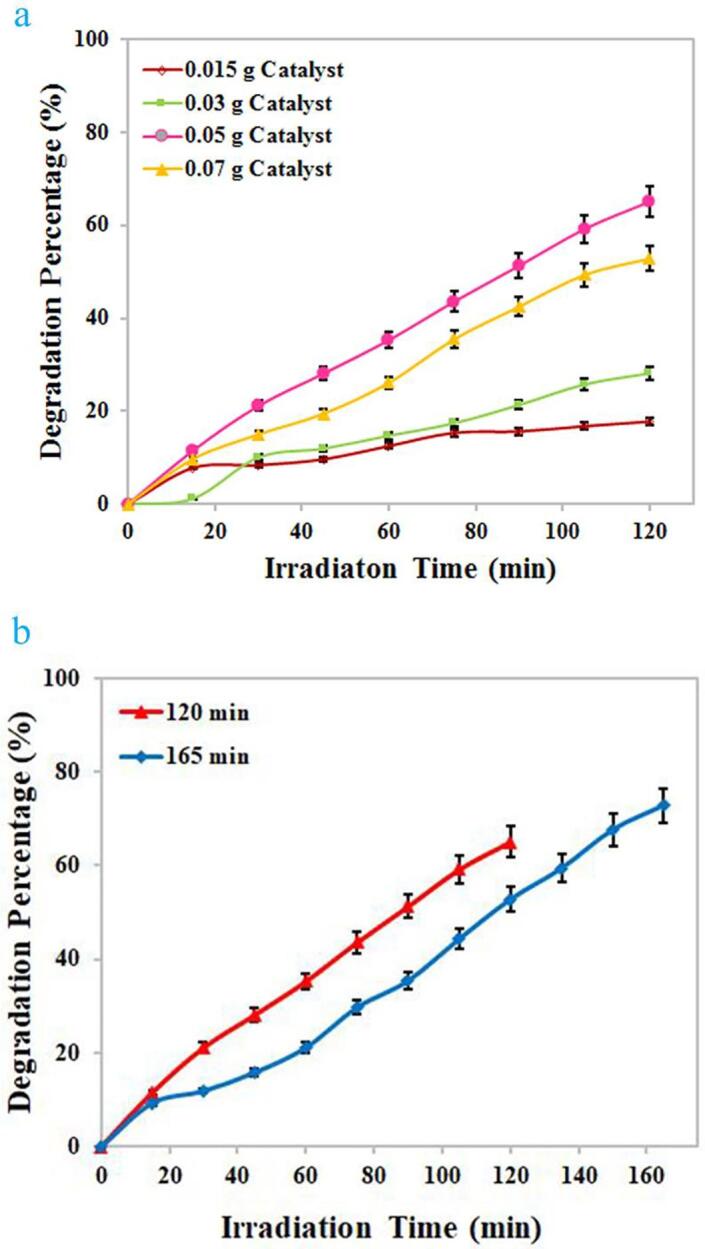
Effects of the quantity of photocatalytic nanostructure (binary Dy_2_O_3_-SiO_2_ nanocomposite) as well as the duration of ultraviolet light on the photocatalytic degradation of Rhodamine B.

Since among the studied contaminants, even in optimal conditions, the lowest percentage of decomposition was observed for Rhodamine B contaminant, in order to achieve a greater percentage of degradation, higher quantities of Dy_2_O_3_-SiO_2_ nanophotocatalyst were employed to degrade the solution with a concentration of 5 ppm (see Fig. 12a). With altering the quantity of Dy_2_O_3_-SiO_2_ nanocomposite from 0.03 g to 0.05 g, an increment in Rhodamine B degradation from 28.16 to 65.07% was observed, and the percentage of Rhodamine B degradation almost doubled. A possible reason for the enhancement in Rhodamine B degradation with increasing nanocatalyst dose could be the increment in the number of active sites as well as the improvement in the adsorption of Rhodamine B molecules on the surface of Dy_2_O_3_-SiO_2_ nanocomposite [58], [59]. However, with a further enhancement in the quantity of Dy_2_O_3_-SiO_2_ nanocomposite to 0.07 g, a decrement in the degradation efficiency of the Rhodamine B was observed, and about 52.82% of the contaminant was degraded. It seems that with the addition in the amount of Dy_2_O_3_-SiO_2_ nanocomposite, owing to the accumulation and precipitation of nanocomposite particles, the light scattering inside the suspension is enhanced, and as a result, the degradation efficiency is diminished [60]. Also, the agglomeration of nanocomposite particles can reduce the number of active photocatalytic sites and be a possible reason for declining the percentage of Rhodamine B decomposition [60]. Thus, the appropriate quantity of Dy_2_O_3_-SiO_2_ nanocomposite for efficient decomposition of Rhodamine B is confirmed, 0.05 g, is confirmed.

Also, to explore the effect of light irradiation time on the enhancement of degradation efficiency, a test was performed in a condition including 0.05 g of Dy_2_O_3_-SiO_2_ nanocomposite and 5 ppm of Rhodamine B for 165 min (see Fig. 12b). It was observed that by prolonging the UV exposure time, the percentage of Rhodamine B degradation could enhance from 54.77 to 61.53%.

**Fig. 13:**
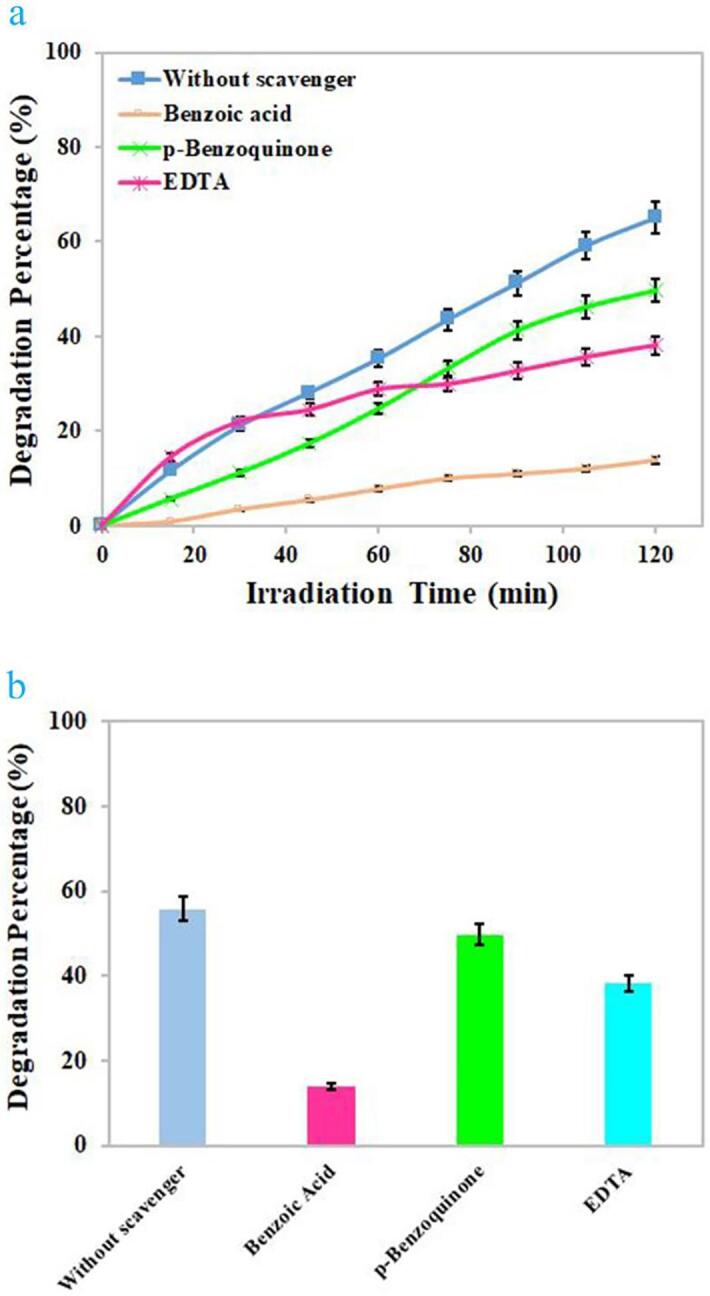
Radical trapping experiment of active species in the photocatalytic degradation of Rhodamine B over binary Dy_2_O_3_-SiO_2_ nanocomposite (sample 5).

**Scheme 2:**
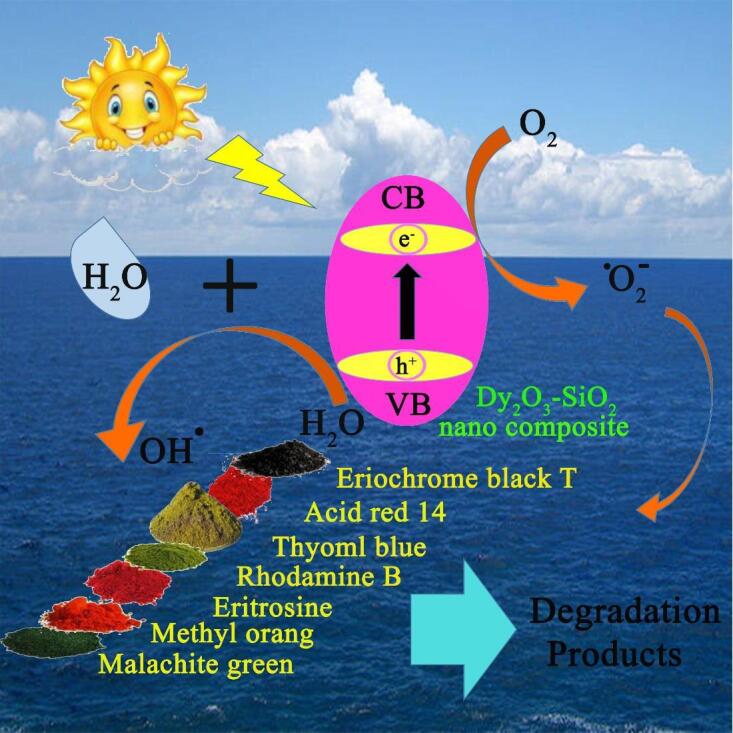
Schematic diagram of the mechanism for the photocatalytic decomposition of various pollutants over binary Dy_2_O_3_-SiO_2_ nanocomposite (sample 5).

The effects of various scavengers upon the photodecomposition of Rhodamine B by binary Dy_2_O_3_-SiO_2_ nanocomposite are illustrated in Fig. 13. Benzoic acid, EDTA, and p-benzoquinone were utilized to quench OH^.^, h^+^, and O_2_^–^, correspondingly [18]. Without the presence of a scavenger, about 54.77% of Rhodamine B molecules were decomposed by the binary Dy_2_O_3_-SiO_2_ nanophotocatalyst. The addition of different scavengers, to varying degrees, prevented the photocatalytic decomposition of Rhodamine B molecules. P-benzoquinone had the least inhibitory effect on the decomposition of Rhodamine B molecules, because in its presence, 49.76% of Rhodamine B could be decomposed. The presence of benzoic acid and EDTA diminished the photocatalytic efficiency to 13.92% and 38.19%, correspondingly, signifying that OH^.^ radicals are the most active degradative species of Rhodamine B molecules. Of course, holes are also involved in the photocatalytic decomposition of Rhodamine B to a lesser degree than hydroxyl radicals [18]. The feasible mechanism involved in the photodecomposition of Rhodamine B molecules is as below [18] (see Scheme 2):

Dy_2_O_3_-SiO_2_ nanophotocatalyst + hν → Dy_2_O_3_-SiO_2_ nanophotocatalyst* + e^−^ + h^+^

h^+^ + H_2_O → OH^.^ + H^+^

2 h^+^ + 2H_2_O → H_2_O_2_ + 2H^+^

H_2_O_2_ → 2OH^.^

e^−^ + O_2_ → O_2_^–.^

O_2_^–.^ + 2OH^.^ + H^+^→ H_2_O_2_ + O_2_

H_2_O_2_ → 2OH^.^

OH^.^ + Rhodamine B molecules → Degradation products [18]

**Fig. 14:**
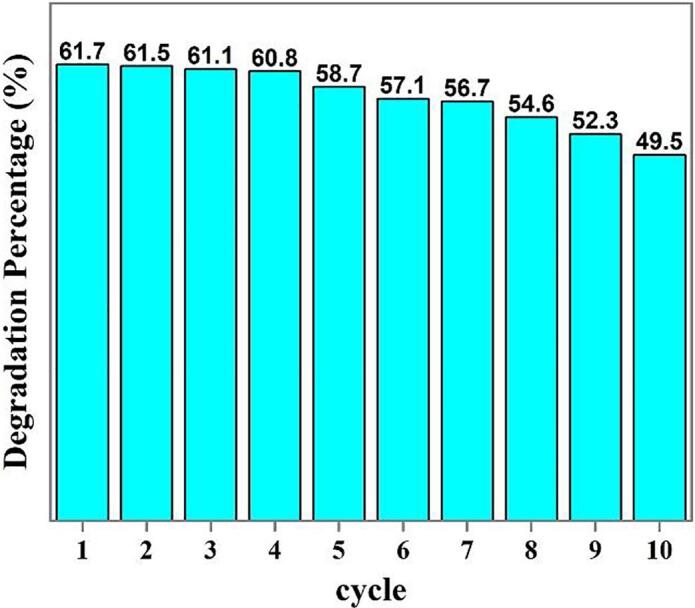
Reusability studies in the degradation of Rhodamine B over binary Dy_2_O_3_-SiO_2_ nanocomposite (sample 5) under UV illumination.

The reusability of the binary Dy_2_O_3_-SiO_2_ nanocomposite (sample 5) was tested for ten cycles in the photodecomposition of Rhodamine B under UV illumination. It was noted that the photodecomposition efficiency diminishes, but was still about 49.5% after the repeated experiments (see Fig. 14).

Table 3 exhibits the photocatalytic efficiency of different compounds for the decomposition of various contaminants under ultraviolet illumination. In this investigation, a new photocatalytic nanocomposite (Dy_2_O_3_-SiO_2_) with enhanced catalytic efficiency toward toxic contaminants was efficiently fabricated employing a basic agent, tetraethylenepentamine (Tetrene), through a simple and quick sonochemical approach. As observed in Table 3, the sonochemically fabricated Dy_2_O_3_-SiO_2_ nanocomposite can compete with other compounds as photocatalysts. We can nominate the porous Dy_2_O_3_-SiO_2_ nanocomposite as a new kind of high-performance nanocatalyst in the field of water remediation and environmental cleaning.

**Table 3:** Comparison of photocatalytic efficiency for decomposition of various pollutants between Dy_2_O_3_-SiO_2_ nanocomposite (sample 5) with other compounds under ultraviolet illumination.

Material	Pollutant	Photocatalytic efficiency (%) & duration of decomposition (min)	Reference
La_2_Sn_2_O_7_ nanoparticles	Erythrosine	84 & 120	[61]
TiO_2_-P25	Methyl orange	90.5 & 180	[62]
TiO_2_ particles	Acid Red 14	88 & 150	[63]
SnO_2_ nanoparticles	Eriochrome black T	77 & 270	[64]
CdS nanostructures	Malachite green	42 & 60	[65]
ZnO-CdO nanocomposite	Thymol blue	46 & 120	[66]
Dy2O3-SiO2 nanocomposite	Thymol blue	81.7 & 120	This work
Dy_2_O_3_-SiO_2_ nanocomposite	Malachite green	71.08 & 120	This work
Dy_2_O_3_-SiO_2_ nanocomposite	Eriochrome black T	77.69 & 120	This work
Dy_2_O_3_-SiO_2_ nanocomposite	Acid Red 14	62.2 & 120	This work
Dy_2_O_3_-SiO_2_ nanocomposite	Methyl orange	71.43 & 120	This work
Dy_2_O_3_-SiO_2_ nanocomposite	Erythrosine	92.99 & 120	This work

## 4 Conclusions

In summary, a new photocatalytic nanocomposite (Dy_2_O_3_-SiO_2_) with enhanced catalytic efficiency toward toxic contaminants was efficiently fabricated employing a basic agent, tetraethylenepentamine (Tetrene), through a simple and quick sonochemical approach. The features of the fabricated photocatalytic nanocomposite were examined employing a variety of microscopic and spectroscopic methods. According to the outcomes of morphological studies demonstrated, it was found that by properly tuning the sonication time and ultrasound power (10 min and 400 W), a porous nanocomposite composed of sphere-shaped nanoparticles with a narrow size distribution can be made. The optimal nanocomposite sample was tested as a nanostructured catalyst for the photodecomposition of several contaminants. The binary Dy_2_O_3_-SiO_2_ nanophotocatalyst demonstrated superior efficiency toward the decomposition of the studied contaminants, and the highest percentage of erythrosine contaminant (about 92.99) was decomposed. Optimization studies for the photocatalytic decomposition of each contaminant illustrated that the best performance could be achieved at a specific amount of contaminant and nanocatalyst. Trapping experiments illustrated that hydroxyl radicals were more effectively involved in the decomposition of contaminant molecules by Dy_2_O_3_-SiO_2_ nanophotocatalyst. The outcomes of this experimental work demonstrate that the addition of silicon dioxide into Dy_2_O_3_ and the sonochemically fabrication of porous Dy_2_O_3_-SiO_2_ nanocomposite brought a new kind of high-performance nanocatalyst in the field of water remediation and environmental cleaning.

### CRediT authorship contribution statement

**Kamran Mahdavi:** Investigation, Methodology, Formal analysis, Software. **Sahar Zinatloo-Ajabshir:** Writing – original draft, Visualization, Writing – review & editing. **Qahtan A. Yousif:** Methodology, Data curation, Writing – review & editing. **Masoud Salavati-Niasari:** Writing – original draft, Writing – review & editing, Conceptualization, Supervision, Project administration, Visualization, Investigation, Methodology, Data curation, Validation, Resources.

## Declaration of Competing Interest

The authors declare that they have no known competing financial interests or personal relationships that could have appeared to influence the work reported in this paper.
